# Supplementary material for: Severity Index for Suspected Arbovirus (SISA): Machine learning for accurate prediction of hospitalization in subjects suspected of arboviral infection
Source: PLoS Negl Trop Dis. 2020 Feb 14;14(2):e0007969. doi: 10.1371/journal.pntd.0007969 (PMC7046343; doi:10.1371/journal.pntd.0007969)
Supplement: S1 Table — Predictors and outcomes are the actual data that are put into the model; for this manuscript the predictors are the variables from each subject and the outcome is whether the subject was hospitalized or not. The final prediction is determined by each algorithm, i.e. the algorithm predicts whether the subject was hospitalized based on the predictor variable values. These final predictions are compared to the true outcome to determine how well each algorithm performed. (DOCX) [file pntd.0007969.s001.docx]

| **Supplementary Table 1: Classification Algorithms Used for Prediction**  Predictors and outcomes are the actual data that are put into the model; for this manuscript the predictors are the variables from each subject and the outcome is whether the subject was hospitalized or not. The final prediction is determined by each algorithm, *i.e*. the algorithm predicts whether the subject was hospitalized based on the predictor variable values. These final predictions are compared to the true outcome to determine how well each algorithm performed. | | | | | |
| --- | --- | --- | --- | --- | --- |
| **Algorithm**  **(Name in Manuscript)** | **Specific Name** | **R Package** | **Type** | **Explanation** | **Further Reading** |
| random forest | - | randomForest | Ensemble, CART | Data are partitioned according to random values of each individual predictor, creating a “decision tree”. This is repeated to create many trees (the “forest”) and final predictions are based on the majority of the predictions made by each individual tree. | [1,2] |
| bagged trees | bagged CART | ipred | Ensemble, CART | Data are divided into subsamples (“bags”) and decision trees are created from each subsample simultaneously. Final predictions are a majority of predictions made by each bag’s trees. See random forest. | [2] |
| k nearest neighbors | - | caret | Instance Based | These models use the outcome data from a selected number (k) of data points (the “neighbors”) and make a final prediction based on the majority prediction of these neighbors. | [1,3] |
| elastic net regression | - | elasticnet | Shrinkage, Regularization | These models shrink the magnitude of correlated coefficients and eliminates correlated predictors from the model then makes final predictions based on the resulting coefficient estimates. | [3,4] |
| generalized boosting models | stochastic gradient boosting | gbm | Ensemble | This model creates a sequential series of decision trees, with each new tree based on the errors of the previous tree. This allows the model to upweight or “boost” the weaker predictions. Final predictions are a combination of all trees. See random forest. | [3,5,6] |
| neural networks | - | nnet | Neural Network | Predictors are treated as “neurons” connected to each other in “networks” with contributing predictors assigned a weight to create a new neuron. Each new neuron is assigned a bias value, and a function is applied to get a prediction. The algorithm adjusts the weights for incorrect predictions as new predictors and neurons are added. All the predictions from these neurons are combined to get the final prediction. | [1] |
| logistic regression | logistic regression with maximum likelihood estimation | - | Regression | This model estimates a logistic relationship between predictors and outcomes; the probability of the outcome is estimated for each set of predictor values. The model finds the coefficient set that will maximize the likelihood of observing the true data. | [1] |

CART=classification and regression trees

**References**

1. Dreiseitl S, Ohno-Machado L. Logistic regression and artificial neural network classification models: a methodology review. J Biomed Inform. 2002;35: 352–9.

2. Breiman L. Statistical Modeling: The Two Cultures. Stat Sci. 2001;16: 199–231.

3. Hastie T, Tibshirani R, Friedman J. The Elements of Statistical Learning: Data Mining, Inference, and Prediction. Second Edition. New York: Springer-Verlag; 2009.

4. Hastie T, Tibshirani R, Wainwright M. Statistical Learning with Sparsity: The Lasso and Generalizations. Chapman & Hall/CRC; 2015.

5. Reid CE, Jerrett M, Petersen ML, Pfister GG, Morefield PE, Tager IB, et al. Spatiotemporal prediction of fine particulate matter during the 2008 northern California wildfires using machine learning. Env Sci Technol. 2015;49: 3887–96. doi:10.1021/es505846r

6. Zhang Z, Zhao Y, Canes A, Steinberg D, Lyashevka O. Predictive analytics with gradient boosting in clinical medicine. Ann Transl Med. 2019;7: 152. doi:10.21037/atm.2019.03.29
