# Supplementary material for: Severity Index for Suspected Arbovirus (SISA): Machine learning for accurate prediction of hospitalization in subjects suspected of arboviral infection
Source: PLoS Negl Trop Dis. 2020 Feb 14;14(2):e0007969. doi: 10.1371/journal.pntd.0007969 (PMC7046343; doi:10.1371/journal.pntd.0007969)
Supplement: S2 Table — The final SISAL model was an elastic net regression model with an alpha value of 0.5, a lambda value of 0.25, and three coefficients (all other coefficients were reduced to zero). (DOCX) [file pntd.0007969.s002.docx]

**Supplementary Table 2: Final SISAL Model**

The final SISAL model was an elastic net regression model with an alpha value of 0.5, a lambda value of 0.25, and three coefficients (all other coefficients were reduced to zero).

| **Variable** | **Coefficient** |
| --- | --- |
| Intercept | 1.34 |
| Drowsiness | -1.46 |
| Retroorbital pain | -0.32 |
| Platelet count | -0.000001 |
