# Supplementary material for: Severity Index for Suspected Arbovirus (SISA): Machine learning for accurate prediction of hospitalization in subjects suspected of arboviral infection
Source: PLoS Negl Trop Dis. 2020 Feb 14;14(2):e0007969. doi: 10.1371/journal.pntd.0007969 (PMC7046343; doi:10.1371/journal.pntd.0007969)
Supplement: S1 Fig — This chart shows the algorithm development, training and testing processes and the flow of data, using an example algorithm with no tuning parameters with the SISA dataset. Repeated 10-fold cross-validation is used for algorithm development to produce an estimate of the final model performance (Mean CV-AUC). The final performance for the algorithm is calculated from the holdout test data. This process was repeated for each algorithm. (DOCX) [file pntd.0007969.s003.docx]

4. Use final algorithm 1 to predict on testing holdout data and calculate final AUC

c. Use preliminary algorithm 1 to make predictions with set-aside 1/10

d. Calculate AUC based on these predictions and the true data for the set-aside 1/10

e. Repeat steps b-d 10 times total, using each 1/10 of the data as the set-aside once, generating 10 AUCs

f. Repeat steps a-e 10 times total

g. Calculated the mean AUC from the 100 AUCs generated

**SISA**

**n=543**

**Training Set**

**n=461**

1. Randomly split data into training (85%) and holdout testing (15%) sets

**Holdout Testing Set**

**n=82**

2. Develop algorithm 1 with training set using repeated 10-fold cross validation

a. Randomly split data into 10 folds

b. Develop algorithm 1 on 9/10 of data

3. Train final algorithm 1 with all training data validation

*Final AUC*

*Performance of algorithm 1*

*Mean CV-AUC*

*Estimated performance of algorithm 1*
