# Supplementary figures and images for: Severity Index for Suspected Arbovirus (SISA): Machine learning for accurate prediction of hospitalization in subjects suspected of arboviral infection
Source: PLoS Negl Trop Dis. 2020 Feb 14;14(2):e0007969. doi: 10.1371/journal.pntd.0007969 (PMC7046343; doi:10.1371/journal.pntd.0007969)

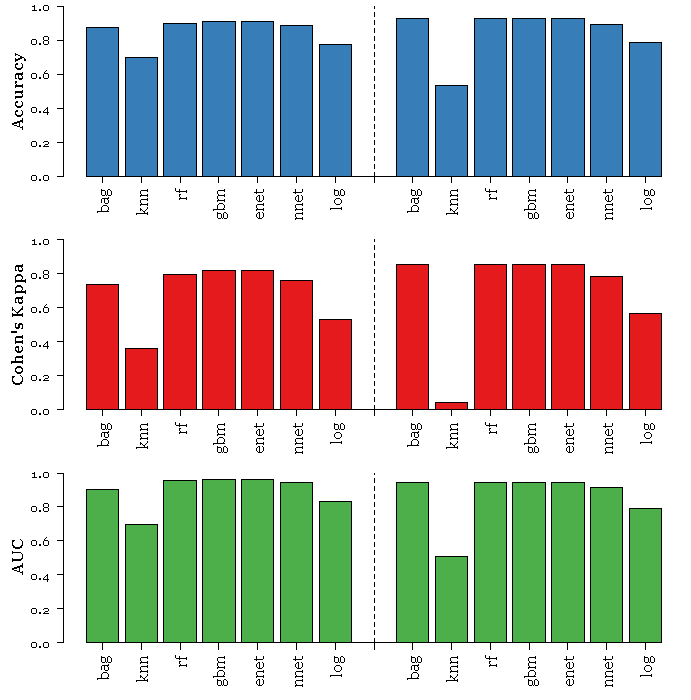


**10-fold Cross-Validation Test Set**

Supplement: S2 Fig — Accuracy (blue), Cohen’s kappa (red), and AUC (green) were calculated for the repeated 10-fold cross validation (left) and the holdout test dataset (right) for prediction of hospitalization status in clinically diagnosed DENV, CHIKV or ZIKV infections. bag = bagged trees, knn = k nearest neighbors, rf = random forest, gbm = generalized boosting models, enet = elastic net, nnet = neural networks, log = logistic regression, DENV = dengue virus, CHIKV = chikungunya virus, ZIKV = Zika virus (DOCX) [file pntd.0007969.s004.docx]
